# PINK1 knockout rats show premotor cognitive deficits measured through a complex maze

**DOI:** 10.3389/fnins.2024.1390215

**Published:** 2024-05-16

**Authors:** Isabel Soto, Vicki A. Nejtek, David P. Siderovski, Michael F. Salvatore

**Affiliations:** Department of Pharmacology and Neuroscience, University of North Texas Health Science Center, Fort Worth, TX, United States

**Keywords:** PINK1 knockout rat, Parkinson’s disease, cognitive, maze, prodromal, trail-making test, locomotor

## Abstract

Cognitive decline in Parkinson’s disease (PD) is a critical premotor sign that may occur in approximately 40% of PD patients up to 10 years prior to clinical recognition and diagnosis. Delineating the mechanisms and specific behavioral signs of cognitive decline associated with PD prior to motor impairment is a critical unmet need. Rodent PD models that have an impairment in a cognitive phenotype for a time period sufficiently long enough prior to motor decline can be useful to establish viable candidate mechanisms. Arguably, the methods used to evaluate cognitive decline in rodent models should emulate methods used in the assessment of humans to optimize translation. Premotor cognitive decline in human PD can potentially be examined in the genetically altered PINK1^−/−^ rat model, which exhibits a protracted onset of motor decline in most studies. To increase translation to cognitive assessment in human PD, we used a modified non-water multiple T-maze, which assesses attention, cognitive flexibility, and working memory similarly to the Trail Making Test (TMT) in humans. Similar to the deficiencies revealed in TMT test outcomes in human PD, 4-month-old PINK1^−/−^ rats made more errors and took longer to complete the maze, despite a hyperkinetic phenotype, compared to wild-type rats. Thus, we have identified a potential methodological tool with cross-species translation to evaluate executive functioning in an established PD rat model.

## Introduction

Parkinson’s disease (PD) is primarily known as a motor disease resulting from dopamine (DA) depletion in the nigrostriatal pathway. However, several non-motor symptoms (NMSs) also significantly impact a PD patient’s quality of life (Postuma and Berg, 2019). One such NMS is cognitive impairment, affecting over one-third of PD patients and presenting up to a decade prior to the onset of motor decline (Durcan et al., 2019). These cognitive impairments can manifest as a subtle decline in executive functioning and typically evade detection by standard global cognitive tests (Dirnberger and Jahanshahi, 2013; Aarsland et al., 2017; Nejtek et al., 2021). Gaining an understanding of the mechanisms underlying prodromal executive function impairments in PD will help identify strategies to delay the onset of motor impairment. This is particularly important as individuals with PD typically seek medical attention only after motor deficits appear (Hauser, 2018).

Phosphatase and tensin homolog-induced kinase 1 (PINK1) is a protein involved in mitophagy; a loss of function mutation within PINK1 is associated with early-onset PD (Valente et al., 2004; Ishihara-Paul et al., 2008; Ricciardi et al., 2014; Schneider and Klein, 2018; Hayashida et al., 2021). The PINK1^−/−^ rat, a genetic preclinical model of early-stage PD, may provide an opportunity to evaluate cognitive impairments that manifest during the premotor phase. Still in its infancy, studies using the PINK1^−/−^ model show some controversy about the age of onset of behavioral symptoms and the extent of these deficits across the lifespan of the rat or mouse models (Dave et al., 2014; Grant et al., 2015; Kelm-Nelson et al., 2021; Salvatore et al., 2022; Soto et al., 2024). Apart from motor dysfunction, one lab has reported vocalization deficits in PINK1^−/−^ rats (Grant et al., 2015), and only one study to date has reported object discrimination deficits at 5 months of age using the novel object recognition (NOR) task as a premotor characteristic in this model (Pinizzotto et al., 2022). We reasoned that a more precise characterization of executive functioning in the PINK1^−/−^ rat could present an important experimental procedure toward establishing its suitability for investigating early biological markers and mechanisms underlying cognitive impairment in PD (Zhang et al., 2022).

The Trail Making Test (TMT) is a goal-directed task used to measure attention, cognitive flexibility, and working memory in humans (Bowie and Harvey, 2006; Nejtek et al., 2021). Our primary goal was to explore whether a rodent task might detect premotor executive functioning deficits that would translate better to TMT outcomes in human PD than other animal testing paradigms (Ferris et al., 2018; Cai et al., 2019). To accomplish this goal, we modified the Cincinnati water maze (CWM) and used it in comparison to the NOR to investigate its ability to provide translatable cognitive outcomes from the PINK1^−/−^ rat that could mirror TMT results in humans with PD.

The CWM measures goal-directed activity and spatial navigation processes that rely on visuospatial function, motivation, and episodic memory associated with the striatum, prefrontal cortex (PFC), and hippocampus (Braun et al., 2012, 2015; Vorhees and Williams, 2016). Similarly, the TMT in humans requires visuospatial acumen and motivation to perform a visual search, which also requires cognitive flexibility. During this task, the PFC and the nigrostriatal dopaminergic pathways are activated (Sawamoto et al., 2008; Nobili et al., 2010; Hofmann et al., 2021). The neurobiological characteristics of the TMT have been examined in prodromal and early-stage PD, and, therefore, the TMT is the ideal translational task to use in a pilot study comparison between the NOR and the modified CWM (Sawamoto et al., 2008). Targeting these neural domains of cognition increases the probability that the preclinical (rodent-based) outcomes will align with established TMT results in human PD. In doing so, we expected to measure episodic memory and visuospatial abilities that are more sensitive to subtle cognitive decline (such as the TMT) than those measuring global cognitive functioning (Garcia-Diaz et al., 2014; Kaul and Elble, 2014; Nejtek et al., 2021; Salvatore et al., 2021; Cammisuli et al., 2022; Mishra et al., 2022).

Here, we modified the CWM to a non-water appetitive 5-T choice-arm (5-T) maze to avoid the anxiety-inducing stress response in rats triggered by swimming that introduces an inherent confound to cognitive outcomes (Harrison et al., 2009; Vorhees and Williams, 2016). Similarly, we chose a modest and transient food restriction of 20% of daily intake for 7 days during the testing period rather than more severe restrictions for an extended period of time, the latter of which could confound interpretations of cognitive performance and necessitate additional controls for such a possibility. For example, some studies have shown that even an acute period of food restriction can significantly interfere with cognitive functioning, but other studies show an improvement (Rajab et al., 2014; Ingram and de Cabo, 2017). Heavily restricting food intake to a degree where body mass is affected could negatively impact cognition or drive some animals to seek the food reward more than others, thus adversely influencing the results (Ingram and de Cabo, 2017). Therefore, we administered an appetitive 5-T maze to 4-month-old PINK1^−/−^ rats under mitigated confounding variables in comparison to NOR task results and determined if components of premotor cognitive decline observed in early-stage PD human subjects could be identified (Nejtek et al., 2021; Mishra et al., 2022).

## Methods

PINK1^−/−^ (*n* = 20) and wild-type (WT) Long Evans (*n* = 14) rats were acquired from Envigo/Inotiv (Boyertown, PA) at 3 months old and were handled daily for 1 week prior to any behavioral assessments. As this is a proof-of-concept study, the sample size was determined from recent motor data using this model (Salvatore et al., 2022). Additionally, we only examined male rats, as over two dozen studies have shown male and female rats perform similarly in preclinical cognitive studies (Becegato and Silva, 2022). Rats were single-housed and kept on a 12-h reverse light–dark cycle with *ad libitum* food and water. All procedures were approved by the Institutional Animal Care and Use Committee (IACUC) at the University of North Texas Health Science Center (UNTHSC) and the ACURO at the Department of Defense.

**Figure 1:**
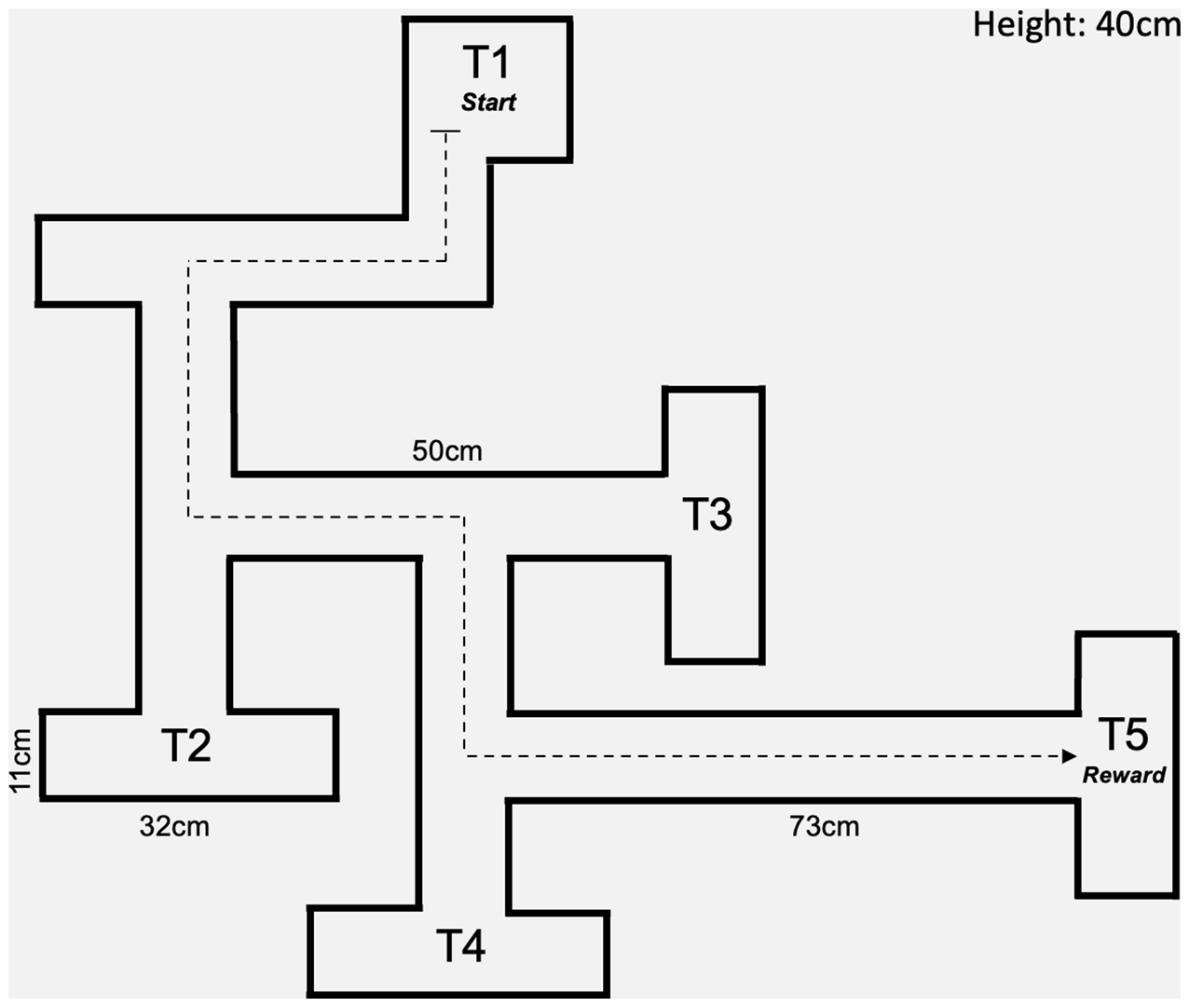
Diagram of maze configuration.

The CWM (MazeEngineers; Skokie, IL) is made of acrylic and consists of multiple arms arranged to produce a maze with different choice arms and one final endpoint. The maze was arranged into simplified “five choice arms” (5-T) following trial runs with pilot rats not part of this studied cohort (Figure 1). The rats were tested on the maze 1 month after their arrival at UNTHSC at 4 months of age. The task was conducted under regular light in the awake cycle in an otherwise empty and bare room (thus no navigational cues were present outside of the maze itself) in two phases: the training phase and the testing phase. During day 1 of the training phase, IACUC chow pellets were scattered inside the maze, and rats were separately given 10 min to explore the area. On the second day, the quantity of scattered food was reduced, and Stauffer’s animal crackers were added to the endpoint. On the third and final day of training, all the scattered food was removed, and crackers and chow pellets were placed exclusively at the final designated arm.

The 5-day testing phase took place 2 days after the conclusion of the 3-day training phase. During the testing phase, each rat underwent one trial per day for 5 consecutive days. *Ad libitum* food intake from rats was reduced by 20% of their regular intake starting the day of completion of the training phase and was maintained during the days of testing. The rats were given 10 min to acclimate to the light and room conditions before being placed in the maze. The rats were then individually video-recorded while the technician remained in the room but away from the maze. Each rat was removed from the maze as soon as the camera screen showed it had reached the endpoint and collected the food. Each rat was allowed a maximum of 5 min to complete the task, and the maze was thoroughly cleaned with disinfectant wipes and dried between uses. Once returned to their home cage, the food trays were filled again to 80% of normal chow pellet consumption. The rats were manually scored by an experimenter blinded to the treatment group on completion time and the number of errors made. Errors constituted any time the rat entered (nose-poke or whole body) an incorrect stem/arm of the maze and/or made any incorrect turns (Vorhees and Williams, 2016).

To use as a comparison to the maze results, the NOR test was conducted when rats were 4 months old on the day following the completion of the maze testing phase. The rats were each placed alone in a 24″ × 24” × 20″ container under red light and allowed to freely explore the area for 10 min. The rats were then removed, and two identical objects were placed in the corners, equally spaced apart from the perimeters of the box. The rats were reintroduced into the testing area immediately and video-recorded for 3 min. After 1 h, one of the initial objects was placed back into the box along with a novel item of similar size but of different colors and materials. The rats were again placed into the box and video-recorded for 3 min. Each rat was evaluated based on the duration of time spent with the novel versus the familiar object. A discrimination index formula [(Novel)/(Novel+Familiar)] was used to evaluate each rat’s ability to differentiate between the objects (Pinizzotto et al., 2022; Soto et al., 2024).

OFT chambers (Columbus Instruments Inc.; Columbus, OH) were used to measure spontaneous locomotor activity in the awake cycle. To determine the impact of motor deficits on cognitive performance in completing the maze, 1 h of motor activity was assessed, both as average distance traveled and average speed, for 3 consecutive days at 1-month pre-maze and 1-week post-maze assessments (i.e., at 3 and 4 months old). The OFT 4-month-old results were also used to measure anxiety by quantifying the average percentage of time spent in the center of the arena compared to the total 1-h period of testing.

### Statistics

GraphPad Prism version 10 was used for statistical analyses. Repeated measures two-way ANOVA and Bonferroni *post-hoc* analysis were used in assessing day-to-day performance in the maze and to compare motor performance between PINK1^−/−^ and WT rats from arrival at 3 months to 4 months old. To determine overall performance on the maze, the mean time to completion and number of errors were calculated for each rat. A Student’s *t*-test analysis was then used to determine time and error differences between the two genotypes at each time point. Student’s *t*-test was also used to analyze differences between genotypes on weight, time spent in the central area of the arena, and the NOR discrimination index. Grubb’s test was used to determine any outliers in OFT, NOR, and average maze scores using the significance level of alpha = 0.05. Only one outlier was identified in the average maze time.

## Results

**Figure 2:**
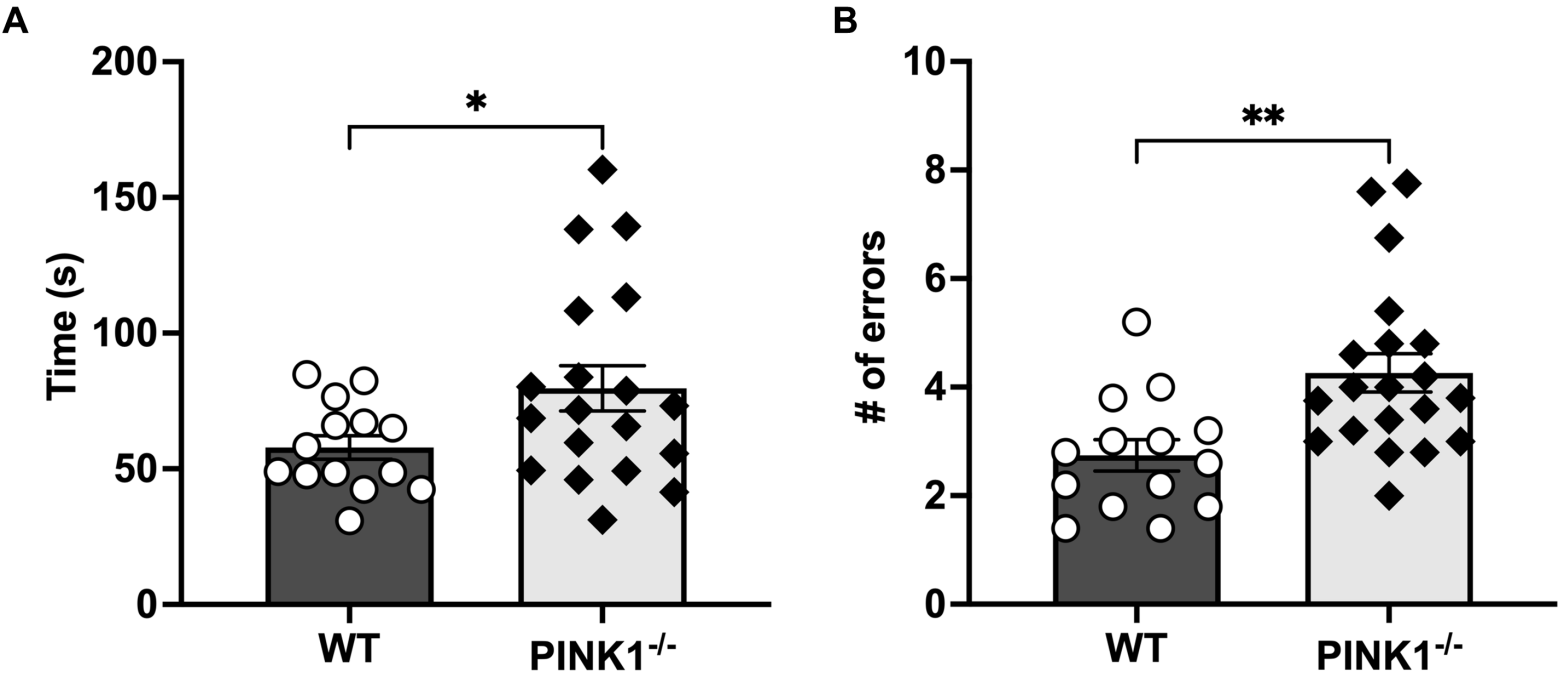
**(A)** Mean maze time to completion. PINK1^−/−^ rats on average of 5 testing days took a significantly longer time to complete the maze than WT controls (*t* = 2.10, *p = 0.04, df = 31). **(B)** Mean maze errors. PINK1^−/−^ rats on an average of 5 testing days made significantly more errors in completing the maze than WT controls (*t* = 3.13, **p = 0.003, df = 32).

**Figure 3:**
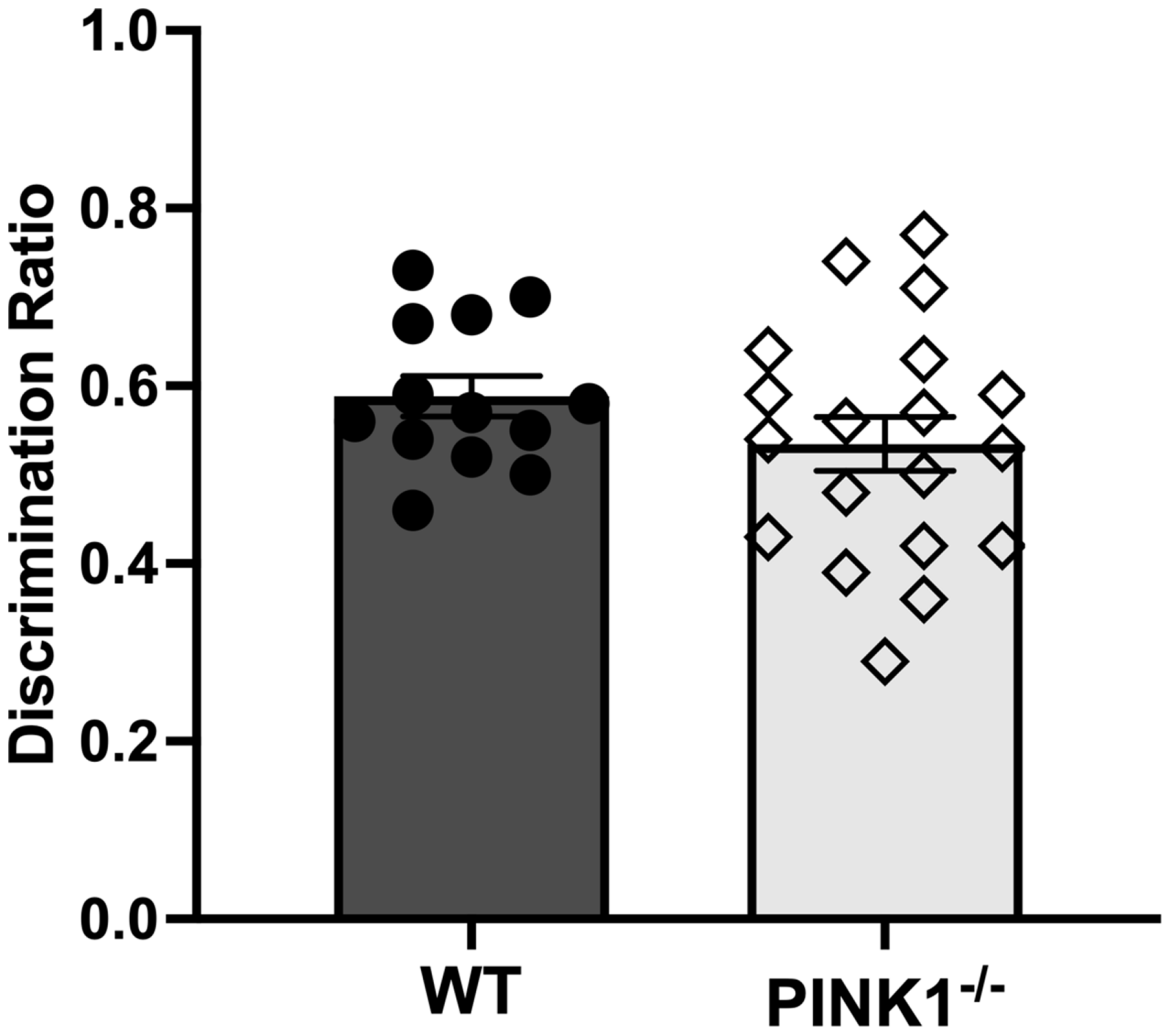
Novel object recognition task. There was no significant difference in the ability to discriminate between a novel and familiar object between WT and PINK1^−/−^ rats at 4 months old. Average Time (s) with objects: WT (Novel: 15.08, sd: 7.93; Familiar: 11.33, sd: 6.29). PINK1^−/−^ (Novel: 15.79, sd: 5.41; Familiar: 13.89, sd: 4.70) (*t* = 0.52, ns, df = 29).

Thirty-four 4-month-old male Long Evans rats (PINK1^−/−^ *n* = 20, WT, *n* = 14) were assessed on latency and the number of errors during the 5-T maze. Two rats were unable to find the endpoint after 5 min on day 1, and only one rat failed in this fashion on day 4 of testing. After calculating average scores, one PINK1^−/−^ rat was determined as an outlier in time to complete the task, and this score was removed from the maze time analysis. Over the five trials, PINK1^−/−^ rats took, on average, a significantly longer time to complete the maze (Figure 2A; WT mean: 58.2, sd: 39.0; PINK1^−/−^ mean: 79.8, sd: 54.69). and committed significantly more errors compared to WT rats (Figure 2B; WT mean: 2.7, sd: 0.89 PINK1^−/−^ mean: 4.26, sd: 0.2). When assessing individual day performance, we did identify a significant genotype effect on maze time to completion [genotype, *F*(1, 32) = 5.01, *p* = 0.03] and errors [genotype, *F*(1, 32) = 9.22, *p* = 0.0047]. Yet, we did not identify a significant difference in trial day performance in either time to completion [trial day *F*(4, 121) = 1.01], not significant (ns); Genotype × trial day [*F*(4, 121) = 1.19, ns] or errors made [trial day *F*(4, 122) = 0.66, ns; Genotype × trial day *F*(4, 122), ns] (Supplementary Figures S1A,B). Notably, we did not find a statistically significant difference in the ability to discriminate between a novel and familiar object in PINK1^−/−^ vs. WT rats (Figure 3). However, we did note that there was considerable heterogeneity in the PINK1^−/−^ results, with ~one-third of the cohort showing worse discrimination index scores.

**Figure 4:**
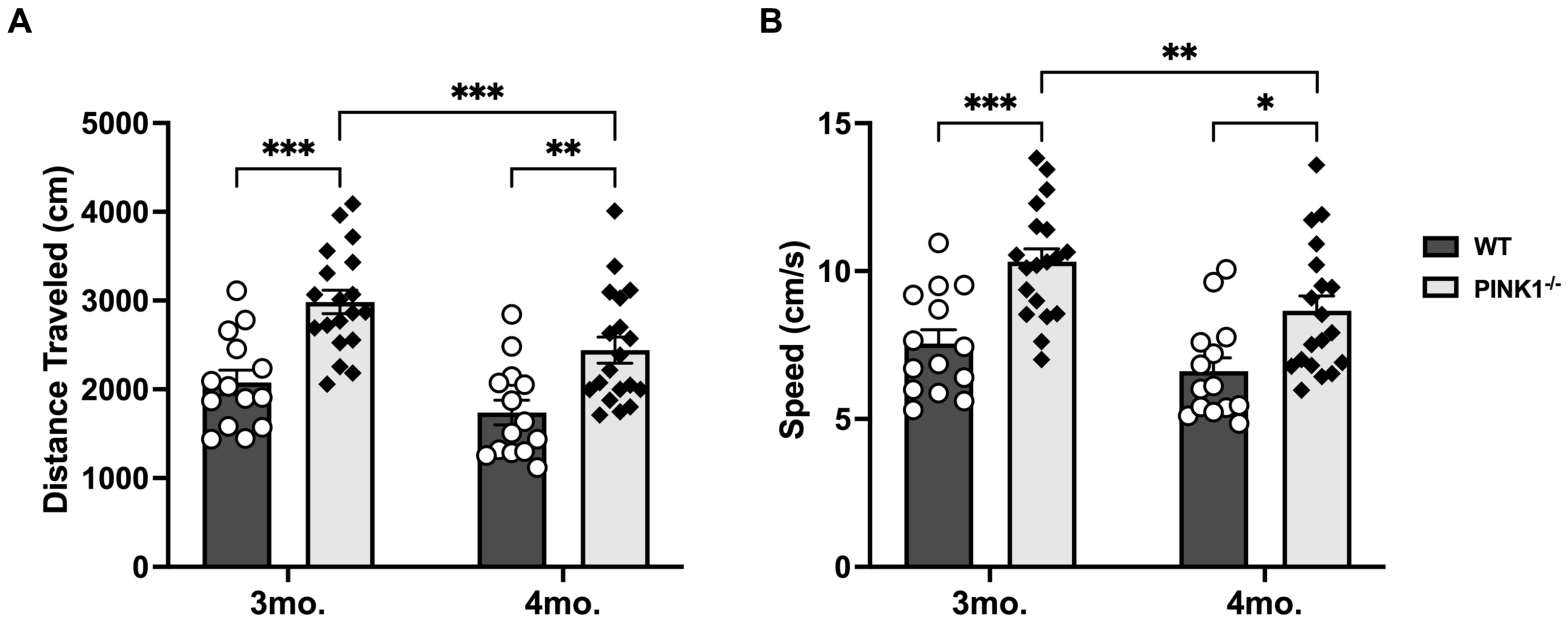
**(A)** Distance traveled in 5 min. Locomotor activity significantly declined in PINK1^−/−^ rats between 3 and 4 months, yet these rats still traveled a significantly greater distance compared to WT rats at both 3 and 4 months old. WT 3mo. vs. 4mo (*t* = 2.31, ns, df = 31); PINK1^−/−^ 3mo. vs. 4mo. (*t* = 4.30, ***p = 0.0006, df = 31); 3mo.WT vs. 3mo. PINK1^−/−^ (*t* = 4.49, ***p = 0.0001, df = 62); 4mo.WT vs. 4mo. PINK1^−/−^ (*t* = 3.48, **p = 0.0036, df = 62). **(B)** Average speed in 5 min. While PINK1^−/−^ rats had a significant decline in average speed between 3 and 4 months, these rats were still significantly faster than WT rats at both 3 and 4 months old. WT 3mo. vs. 4mo (*t* = 1.96, ns, df = 31); PINK1^−/−^ 3mo. vs. 4mo. (*t* = 4.08, **p = 0.0012, df = 31); 3mo.WT vs. 3mo. PINK1^−/−^ (*t* = 4.11, ***p = 0.0005, df = 62); 4mo.WT vs. 4mo. PINK1^−/−^ (*t* = 3.48, *p = 0.01, df = 62).

Considering PINK1^−/−^ rats eventually develop motor dysfunction, spontaneous locomotion was evaluated using the open-field test (OFT) to address the possibility that the longer navigation time in the PINK1^−/−^ rats was related to decreased locomotor activity (Dave et al., 2014; Grant et al., 2015; Salvatore et al., 2022; Soto et al., 2024). Rather than a decrease, PINK1^−/−^ rats displayed hyperactivity during both pre- and post-cognitive testing compared to WT rats, with a significant age and genotype effect in distance traveled (Figure 4A) [Genotype, *F*(1, 31) = 20.67, ****p < 0.0001], Age [*F*(1, 31) = 20.73, ****p < 0.0001], Genotype × Age [*F*(1, 31) = 1.09, ns], and average speed (Figure 4B) [Genotype *F*(1, 31) = 16.18, ***p = 0.0003], Age [*F*(1, 31) = 17.21, ***p = 0.0002]. Genotype × Age [*F*(1, 31) = 1.37, ns] compared to WT rats in the first 5 min or a full hour of OFT (*data not presented*). We also found that PINK1^−/−^ rats spent on average a higher percentage of the 1 h OFT time in the center of the arena compared to WT rats, suggesting a lower level of anxiety (Supplementary Figure S2; WT mean: 19.51, sd: 8.49; PINK1^−/−^ mean: 29.44, sd: 13.88). Moreover, we found no significant difference in average body weight between the two groups, which could add a confounding variable (WT mean: 472.8, sd: 33.8; PINK1^−/−^ mean: 496.2, sd: 45.87 t = 1.41, ns, df = 32).

## Discussion

Our results reveal that PINK1^−/−^ rats take a significantly longer time to complete the maze, despite hyperkinetic locomotor function. They also make significantly more errors than WT rats. These cognitive outcomes align with prior investigations in PINK1^−/−^ rodents, which exhibit deficits in memory and learning capacities (Maynard et al., 2020; Pinizzotto et al., 2022). The 5-T maze requires rats to recall which paths they must travel from the beginning to the end with minimal errors throughout the “five choice arms” configuration. In the TMT test, human subjects with PD must also use working memory and processing speed to connect randomly placed letters and numbers in ascending order on a sheet of paper, aiming to complete the task correctly as quickly as possible. Therefore, this multiple T-maze has a suitable translation to human PD assessments. The more commonly used NOR is not a goal-directed task, and the outcomes are highly dependent on the properties of the objects used (Ennaceur, 2010; Soto et al., 2024). This is further supported by the NOR results in this study. Unlike the maze results, the NOR did not detect a significant difference between the two genotypes. This suggests that the PINK1^−/−^ model may exhibit subtle cognitive deficits specifically in areas of spatial learning as opposed to recognition memory. Therefore, a more complex task than the NOR, such as the 5-T maze used in this study, might be more effective in pinpointing cognitive deficits within the PINK1^−/−^ model.

Our results underscore the potential of utilizing an appetitive-based maze under minimal food restriction to detect cognitive deficits in the earliest stages of decline prior to the onset of motor dysfunction, which may otherwise go unnoticed. The longer time for completion and frequency of errors in PINK1^−/−^ rats indicate that this PD model may be deficient in spatial learning, egocentric navigation, and/or episodic memory in the premotor phase. The largest difference in scores between genotypes was on testing days 1 to 3. Considering the 2-day period between final training and the start of the testing phase, the results suggest that the 4-month-old PINK1^−/−^ rats have both learning and memory consolidation deficits—a neurological symptom that is not a characteristic observed in their age-matched WT counterparts. Although we identified overall differences in genotype performance, we did not identify clear learning acquisition to the maze path across the 5 consecutive trial days in either genotype. This suggests that learning acquisition likely began during the initial 3-day training phase where rats were first exposed to the maze. In fact, learning acquisition has been reported in other studies by the third day of training (Pistell and Ingram, 2010; Pistell et al., 2012; Durán et al., 2023). We speculate that the observed behavior on days 4 and 5 could have resulted from a decreased intrinsic interest in the ongoing food reward, leading rats to choose maze exploration over obtaining the food reward (Durán et al., 2023). Considering the low number of errors made in the testing phase, a more complex configuration of the maze may be able to discern differences in acquisition learning between the genotypes.

Cognitive decline in PD, both in sporadic and familial cases, represents an early symptom that significantly compromises the quality of human life, yet it remains largely unresponsive to currently available pharmacological treatments (Dirnberger and Jahanshahi, 2013; Aarsland et al., 2017; Schneider and Klein, 2018; Fang et al., 2020; Nejtek et al., 2021). A better understanding of the neuro-pathological changes that underlie such deficits will allow a path toward new treatment development and a possible delay in disease progression. The major issue at present is to recognize that this specific decline occurs in the prodromal or in the earliest stage of PD and manifests in a heterogeneous manner as subtle alterations in executive functioning that include cognitive flexibility, decision-making, working memory, attention, or visuospatial abilities (Dirnberger and Jahanshahi, 2013; Kehagia et al., 2013; Ricciardi et al., 2014; Aarsland et al., 2017; Schneider and Klein, 2018; Fang et al., 2020; Hayashida et al., 2021; Nejtek et al., 2021).

Our implementation of the preclinical 5-T maze under limited added stressors, such as an extended period of food restriction, water motivation, or foot shock (Pistell and Ingram, 2010; Pistell et al., 2012), appears to capture the underlying neurobiology of subtle decline in these specific cognitive domains. This increases the likelihood of capturing relevant neurobiological deficits in affected areas, such as the PFC, that are most responsive and most vulnerable to disease progression (Kehagia et al., 2013; Fang et al., 2020; Mishra et al., 2022). Our results suggest that the PINK1^−/−^ rat can serve as an appropriate preclinical model for investigating the early neurobiological mechanisms associated with cognitive functioning also observed in human PD (Ferris et al., 2018; Kelm-Nelson et al., 2021; Pinizzotto et al., 2022; Zhang et al., 2022; Soto et al., 2024).

This is the first study, to our knowledge, to explore the PINK1^−/−^ rat’s capability to complete a food-incentivized maze during the premotor phase. In line with the findings of this study, prior cohorts of PINK1^−/−^ rats in our laboratory have consistently exhibited hyperkinetic behavior before the age of 6 months (Salvatore et al., 2022; Soto et al., 2024). Notably, these outcomes contrast with reports from other studies, where the timing of initial motor deficits and the extent of DA loss in this model range widely, with some indicating no deficits and others observing deficits emerging between 4 and 8 months (Dave et al., 2014; Grant et al., 2015; de Haas et al., 2019; Salvatore et al., 2022; Soto et al., 2024). This period of observed hyperkinesia may be attributed to a compensatory phase preceding symptomatic DA loss, potentially reflecting the premotor phase of PD (Blesa et al., 2017; Creed et al., 2021; Kasanga et al., 2023; Soto et al., 2024). While other types of motor tests in rats may reveal specific deficits, such as in gait, the OFT used here assesses both spontaneous movement and speed, two motor aspects critical to maze completion. As PINK1^−/−^ rats exhibited a hyperkinetic phenotype, it is unlikely that motor impairment can explain their greater time taken to complete the 5-T maze. Furthermore, this observed behavior in the PINK1^−/−^ rats, marked by an increased percentage of time spent in the center of the OFT arena and a higher number of errors in the maze compared to WT rats, indicates that the rats did not merely freeze during the maze assessment. This implies that anxiety did not significantly influence our findings (Lezak et al., 2017). Additionally, the PINK1^−/−^ rats did not exhibit any other prototypical behaviors associated with anxiety, thereby ruling out anxiety as a confound.

While this study did not assess neurobiological differences associated with general, NMSs, brain imaging studies of PINK1^−/−^ rats have found decreased anisotropy in the olfactory system, hypothalamus, thalamus, nucleus accumbens, and cerebellum at postnatal weeks 12–13 (Ferris et al., 2018). A study using resting-state functional MRI identified reduced connectivity between the neostriatum, midbrain dopaminergic regions, hypothalamus, and thalamus and increased connectivity between the ventral midbrain dopaminergic regions and hippocampus in 6–8-month-old PINK1^−/−^ rats compared to WT (Ferris et al., 2018; Cai et al., 2019). Still, a study measuring hippocampal synaptic plasticity between 4 and 5 months old found no impairment in PINK1^−/−^ rats compared to controls (Memon et al., 2021). Our study in this PD rat model showed elevated levels of both norepinephrine (NE) and DA in the PFC at a younger age, suggesting the possibility of abnormal DA or NE signaling therein (Soto et al., 2024). This indicates that future studies are required to assess mechanisms in the PFC during this stage, as well as the progression of this cognitive phenotype, as some studies suggest that early cognitive decline may be indicative of disease trajectory (Fang et al., 2020). Additionally, it would be beneficial if PINK1^−/−^ rats are able to navigate more complex paths in the maze, which may better assess cognitive flexibility vs. memory capacity.

A limitation to consider is the unclear influence of olfaction on our study results, given that hyposmia is an early indicator of PD (Sui et al., 2019). The research on how olfaction is affected by the PINK1^−/−^ mutation is limited and has mixed results. While Cai et al. (2019) found reduced olfactory bulb size and connectivity in rats aged 6–8 months, Ferris et al. (2018) found that rats aged 13–14 weeks retained normal sensitivity to new odors. Therefore, it is unclear if, at 4 months old, PINK1^−/−^ rats would exhibit significant behavioral changes related to olfaction. However, the overall low error rates observed across both genotypes in this study suggest that memory recall rather than exploratory behavior driven by scent may be the dominant factor influencing our results.

Finally, as this research was a proof-of-concept design, only male rats were tested to reduce the sample size needed because studies have shown that male PD patients often exhibit more pronounced cognitive impairments compared to female PD patients. To determine if such distinctions exist in this rat model, future investigations should include an evaluation of sex differences to offer a more comprehensive understanding (Reekes et al., 2020).

Overall, our results show that male PINK1^−/−^ rats are compliant and capable of participating in a complex cognitive task and yet show potential signs of prodromal cognitive deficits comparable to executive function impairments seen in prodromal and early-stage individuals with PD. This outcome provides a rationale to evaluate whether catecholamine function and related signaling pathways are compromised in the PFC and hippocampus prior to hypokinesia onset in order to further delineate mechanisms that compromise specific components of cognition.

## Data availability statement

The raw data supporting the conclusions of this article will be made available by the authors, without undue reservation.

## Ethics statement

The animal study was approved by the Institutional Animal Care and Use Committee (IACUC) at the University of North Texas Health Science Center (UNTHSC). The study was conducted in accordance with the local legislation and institutional requirements.

## Author contributions

IS: Writing – review & editing, Writing – original draft. VN: Writing – review & editing, Writing – original draft. DS: Writing – review & editing, Writing – original draft. MS: Writing – review & editing, Writing – original draft.
