# Supplementary material for: PINK1 knockout rats show premotor cognitive deficits measured through a complex maze
Source: Front Neurosci. 2024 May 16;18:1390215. doi: 10.3389/fnins.2024.1390215 (PMC11137248; doi:10.3389/fnins.2024.1390215)
Supplement: Supplementary file 1 [file Data_Sheet_1.pdf]

Supplemental Figures

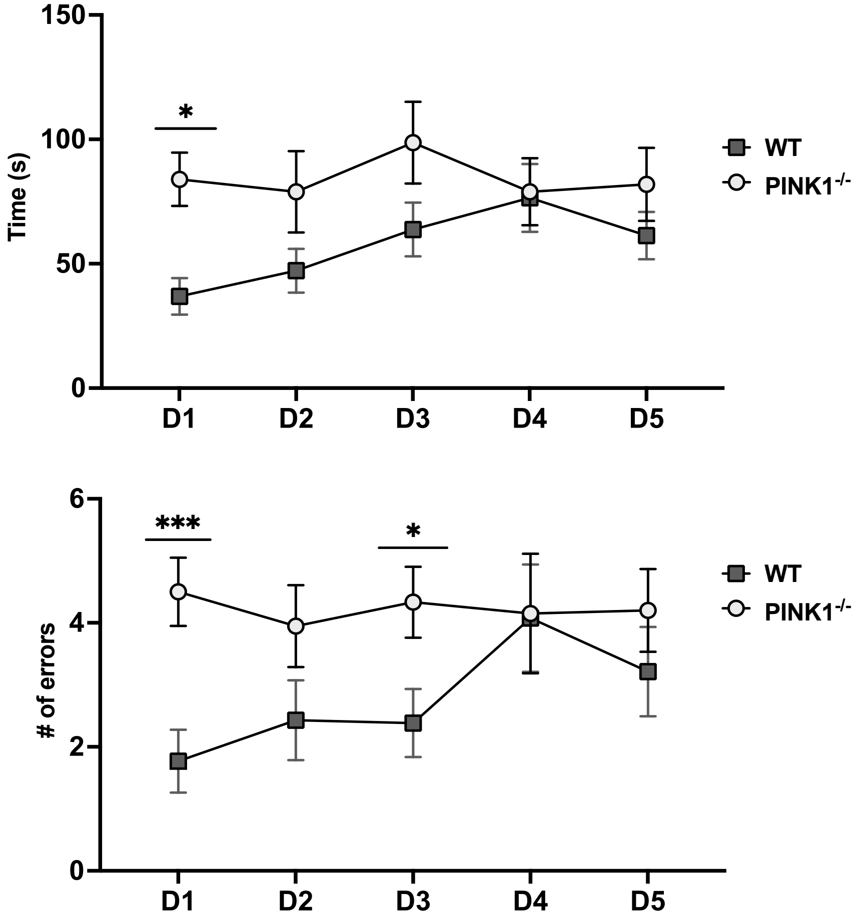

Suppl. Fig 1A. Multiple T-Maze Day by Day Average Time to Completion. $PINK1^{-/-}$ rats took a significantly longer time to complete the maze compared to WT rats specifically on day 1, with a trend towards significance in day 2 and 3. There was no significant effect of trial day. WT vs $PINK1^{-/-}$: Day 1 (t= 2.58, **p*= 0.01, df= 32); Day 2 (t= 1.76, *p*= 0.08, df= 32); Day 3 (t= 1.76, *p*= 0.08, df= 32); Day 4 (t= 0.16, *p*= 0.87, df= 32); Day 5 (t= 1.07, *p*= 0.29, df= 32).

Suppl. Fig 1B. Multiple T-Maze Day by Day Average Errors. $PINK1^{-/-}$ rats made significantly more errors reaching the end of the maze compared to WT rats specifically on days 1 and 3. There was no significant effect of trial day. WT vs $PINK1^{-/-}$: Day 1 (t= 3.65, ****p*= 0.001, df= 32); Day 2 (t= 1.65, *p*= 0.11, df= 32); Day 3 (t= 2.46, **p*= 0.02, df= 32); Day 4 (t= 0.05, *p*= 0.96, df= 32); Day 5 (t= 1.00, *p*= 0.32, df= 32).

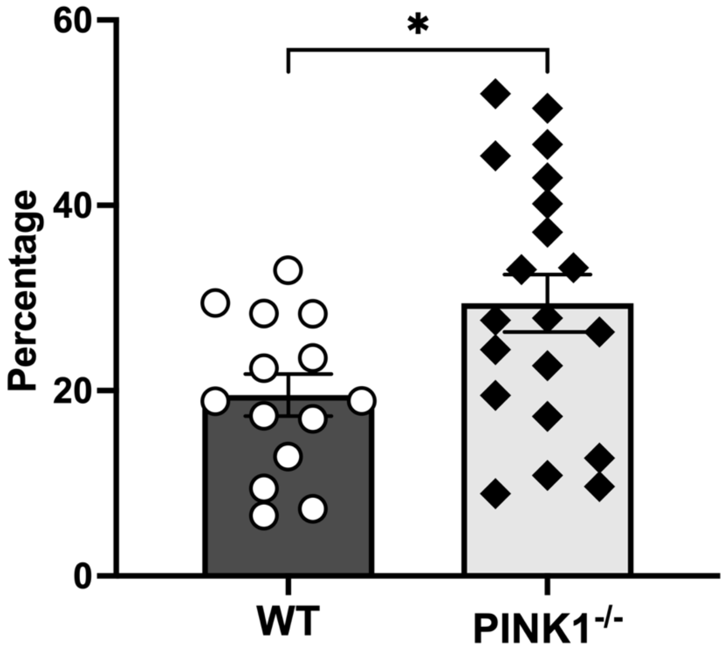

Suppl. Fig 2. Anxiety. PINK1$^{-/-}$ rats spent a significantly higher percentage of the one-hour period in the the center of the arena during the Open Field Test compared to WT control rats, which is indicative of lower levels of anxiety. (t= 2.38, *p= 0.02, df= 32)
